# Point-of-care core needle biopsy pathway for early diagnosis of lymph node masses: comparative costing of a scalable pathway

**DOI:** 10.1186/s13561-026-00715-1

**Published:** 2026-02-06

**Authors:** David Richardson, Waarisa Fareed-Brey, Katherine Antel, Karryn Brown, Jenna Bailey, Estelle Verburgh, Lucy Cunnama

**Affiliations:** 1 Division of Clinical Haematology, Department of Medicine, Faculty of Health Sciences, University of Cape Town and Groote Schuur Hospital, Cape Town, South Africa; 2 Health Economics Unit and Division, School of Public Health, University of Cape Town, Anzio Road, Cape Town, South Africa; 3 Department of Hematology Oncology, Faculty of Medicine, Medical University of South Carolina, Charleston, USA

**Keywords:** Lymphadenopathy, Core needle biopsy, Fine needle aspirate, Surgical excision biopsy, Comparative cost analysis, Diagnostic pathways, Lymphoma, Tuberculosis

## Abstract

**Background:**

There are three common and significant causes of lymphadenopathy (LAP) in tuberculosis-and-HIV-endemic settings - tuberculosis, lymphoma, and metastatic solid organ cancers. Overlapping clinical symptoms of these diseases necessitates diagnostic procedural investigation. Fine needle aspiration (FNA) with Xpert TB/RIF Ultra has good sensitivity for tuberculosis diagnosis, while FNA cytology can detect solid cancer lymph node metastasis, although with lower sensitivity. Critically, lymphoma cannot be reliably detected or classified using FNA. Lymphoma diagnosis often is made after repeat testing and eventual surgical excision biopsy (SEB), contributing to treatment delays, inappropriate empiric anti-tuberculosis therapy, and increased strain on already limited healthcare resources.

**Methods:**

This study presents a comparative provider-perspective cost analysis of three diagnostic procedures: FNA, SEB and core-needle biopsy (CNB). Costs were evaluated for individual procedures and within investigative pathways for unexplained LAP. Data were collected at Groote Schuur Hospital, a tertiary academic hospital in South Africa, during 2018 and 2025. Costing was conducted using a mixed-methods approach combining bottom-up ingredients-based costing with top-down allocation of overheads. Capital costs were annualised, and all costs were adjusted to 2025 values in United States dollars.

**Results:**

Histological diagnosis and tuberculosis testing using CNB were less costly (US$ 145) than either SEB (US$ 244), or FNA cytology and tuberculosis testing (US$ 179). Total investigative costs increased substantially when repeat FNAs were required while awaiting access to SEB (US$ 518). CNB demonstrated high diagnostic accuracy and obviated the need for further procedures in most cases, improving diagnostic efficiency and offering significant potential for cost savings. Sensitivity analyses suggested further reductions in CNB costs are possible through task-shifting to junior clinicians and through the use of reusable biopsy equipment.

**Conclusion:**

The CNB-centred diagnostic approach to lymphadenopathy provides lower-cost, timeous, and accurate diagnosis of the three most significant causes of LAP in tuberculosis- and HIV-endemic settings. This approach enables early initiation of appropriate treatment, reduces unnecessary procedures, and optimises the use of limited healthcare resources. These findings support the integration of CNB into national diagnostic algorithms and highlight the value of investments in training and equipment to enable its decentralised implementation.

## Introduction

### The growing burden of cancer

Cancer is projected to become the leading cause of mortality in every country within this century as its incidence and mortality are increasing worldwide [1]. Lymphoma, cancer of the lymphatic system, is amongst the top ten most frequently occurring cancers globally and is emerging as the principal cause of cancer mortality in people living with HIV (PLWH) who are at significantly increased lymphoma risk [2–4]. Importantly, lymphoma also ranks among the top ten cancers that can be cured over time, making early diagnosis essential and potentially lifesaving [2–5].

Lymphomas are broadly classified as non-Hodgkin’s Lymphoma (NHL) or Hodgkin Lymphoma (HL) based on the tumour’s microscopic appearance (or histology), and can be classified into three clinical groups based on typical disease behaviour: low-grade (or indolent), high-grade (or aggressive), and very high grade [6, 7]. Even with antiretroviral therapy (ART), PLWH remain at a more than 10-fold increased risk of lymphoma, with aggressive and very-aggressive sub-types, such as Burkitt lymphoma and diffuse large B-cell lymphoma being disproportionately increased [4].

Lymphadenopathy (LAP)—an abnormal enlargement of lymph nodes (LNs)—is often the first sign of lymphoma. However, the differential diagnoses for LAP, includes other malignancies (such as leukaemia, Kaposi’s sarcoma, skin cancer, and cancer metastases), as well as extrapulmonary tuberculosis (EPTB), other infections, drugs, and autoimmune conditions [8, 9]. Obtaining an expedited diagnosis is critical and doctors must investigate patients - in a pragmatic, cost-conscious manner - that can efficiently prove, or exclude, tuberculosis infection and common and important differential diagnoses – principally, lymphoma and disseminated malignancy [8, 10]. However, in tuberculosis-endemic areas, like South Africa, EPTB is the commonest cause of LAP and the focus of diagnostic investigations [11, 12].

### Investigation of LAP in a tuberculosis-endemic environment

Tuberculosis was second only to the coronavirus as a single infectious disease cause of death in 2022 and continues to be a critical health challenge [13, 14]. Definitive diagnosis of EPTB is, however, challenging. Mycobacterial culture is time-consuming and diagnostic yield is limited by difficulty accessing diagnostic tissue and the pauci-bacillary nature of the disease [15]. Likewise, poor adoption of Xpert TB/RIF Ultra (Ultra), and other nucleic acid amplification tests, with persistent use of low-yield techniques such as staining for acid-fast bacilli on fine needle aspirate (FNA), leads to frequent false negative results in the work-up of patients with LAP in whom tuberculosis is suspected [11, 16].

This risk is mirrored by the frequent use of FNA cytology (the study of individual cells) to investigate for lymphoma and other cancers in patients presenting with easily accessible and palpable LN [17]. For lymphoma diagnosis, the sensitivity of FNA cytology in our context has been shown to be unacceptably low, as low as 11% [18]. This is supported by international literature and likely reflects the poor performance of FNA cytology in detecting high-grade, or aggressive, lymphomas which are more frequent in HIV-endemic settings [4, 19–21]. In addition to missed diagnoses, even in the cases where lymphoma is detected, definitive diagnosis is not given, but rather cases are reported as being “atypical”, “suspected malignancy”, or a “lymphoproliferative disorder” [22]. Ancillary investigations are then required for precise diagnoses that can inform treatment [6, 23, 24]. These limitations of FNA cont**r**ibute to the expert recommendation for surgical excision biopsy (SEB) with histology (the study of cells and structures in tissue to aid diagnosis) rather than FNA cytology, as the ideal cancer diagnostic tool [23]. The ultrasound-guided core needle biopsy (CNB) is increasingly recognised as an alternate diagnostic tool – of particular benefit when LNs are inaccessible or when SEB is not readily available [18, 25–27]. Despite this recommendation, FNA cytology is often preferred in low-resource and tuberculosis-endemic settings for younger patients as it is less costly and easier to administer at a primary care level [19, 24].

Consequent to these inappropriate test selections, first-line LAP investigations are commonly non-diagnostic. Patients then undergo repeat testing, are referred for SEB or, in tuberculosis-endemic areas, their constitutional symptoms - fever, night sweats, and weight loss - are ascribed to EPTB and patients are commonly placed on empiric tuberculosis therapy. This increases the risk of misdiagnosis and treatment delays in patients with lymphoma and other malignancies in which these symptoms are also present (see Table 1) [9, 29, 30].

**Table 1:** Confounders of typical lymphoma ‘presentation syndromes’

Signs and Symptoms	Presentation	Confounders/Differential Diagnosis
B -symptoms *Triad described in presentation*	Unexplained temperature > 38 °C, drenching night sweats, and > 10% weight loss in the last six months.	Constitutional symptoms are non-specific disease features seen in numerous other conditions. These include HIV, TB, connective tissue disease, solid organ cancers, and endocrine conditions.
Significant LAP	Non-painful LNs that are frequently symmetrical and of rubbery, hard texture. Typically present for > 3 weeks and > 1,5 cm in size (although smaller LN are seen in lymphoma)	Other conditions that present with peripheral LNs that can confound the diagnosis of lymphoma include: viral conditions like HIV or herpes; bacterial infections like Staphylococcus aureus, TB; malignancy, and connective tissue disease.
Cytopenia	Lymphomas invade the bone marrow and suppress normal cell production and commonly result in low peripheral blood counts, especially anaemia	Other haematological confounders include acute myeloid leukaemias (AML) or myelodysplastic syndrome (MDS). The differential diagnosis for cytopenia is wide and traverses across many systematic and immunological conditions.
Lymphocytosis	Due to the spill of tumour cells into the bloodstream, typically after bone marrow invasion. These blood-borne cells are easily diagnosed as cancer cells.	One of the pitfalls of diagnosing lymphoma is that differential blood counts are often not requested. It is recommended that a full blood count with a differential count and blood film should be requested in any patient with an abnormal point of care haemoglobin.
Tumour masses	Lymphoma masses grow indolently in body cavities and later cause dysfunction. This may manifest as a mediastinal mass with compressive effects, pleural effusions, or gastrointestinal obstruction, as is often seen in B cell lymphomas.	Histological diagnosis is the key to establishing the cause of the tumour mass and is supported by imaging. In young patients, lymphoma is the primary cause of mediastinal masses and is an important differential diagnosis of lung cancer in all ages. In TB endemic areas, patients presenting with pleural effusions are commonly commenced on empiric TB treatment, which is acceptable, however, it is recommended that they are followed up two weeks later with lymphoma being considered as the differential diagnosis.

Adapted with permission from Verburgh, et al. (2019) [28]

*LN* Lymph node, *TB *Tuberculosis

### Understanding the cost of LAP investigation pathways

Over the last few years, economic evidence has been published for lymphoma treatment and management. Papers have included evaluations of the cost, life expectancy and quality of life in patients with lymphoma [31], real-world evaluation lymphoma treatment options, including using Rituximab [32–34], and the cost of frontline treatment failure [33]. In 2018, Bosch et al. published a retrospective study of 1 779 patients, which assessed time-to-diagnosis and the associated cost in individuals diagnosed with HL, large B-cell lymphoma, and peripheral T-cell lymphomas [35]. The authors found that the time-to-diagnosis was shorter among inpatients (12.3 days) than among outpatients (16.2 days). However, by using bottom-up costing they showed a higher mean cost associated with inpatient care (US$ 5 097 compared to US$ 1 776 for outpatient care – inflated to 2025 US$) [35]. Whilst the shorter time interval may benefit patients with lymphoma as they could receive treatment earlier, the decision-makers would have to reflect on the significant cost implications given resource limitations, especially in low- and middle-income countries (LMICs).

Although numerous studies have addressed the various diagnostic methods for LAP (see Table 2), as well as lymphoma [33, 34], a significant gap exists in comparing the cost of different diagnostic pathways. Locally, Antel (2021) recommended an evolving, novel pathway for rapid lymphoma diagnoses in a tuberculosis-endemic setting. The pathway uses clinical features to determine which diagnostic tests to perform and rapidly progresses to LN biopsy, thus expediting tuberculosis exclusion and shortening the time-to-diagnosis in patients with lymphoma [36]. This method has been shown to have a high diagnostic yield and shorten time to diagnosis, theoretically reducing late lymphoma presentation and thus patient morbidity and mortality [18, 30]. The cost of the diagnostic algorithm is, however, lacking.

**Table 2:** Comparison of the different investigative methods of LAP

FNA	CNB/USCNB	SEB
A small gauge needle is used to aspirate tissue for cytology only -distorts sampling tissue architecture	A larger gauge needle is used to allow histological assessment - retains tissue sample architecture	The diagnostic gold-standard. The whole LN is removed - retains tissue sample architecture
Limited diagnostic yield	Better diagnostic yield than FNA cytology	Highest diagnostic yield
Minimally invasive and simple technique	Minimally invasive and simple technique	More invasive and complex technique
Quickly performed as a point-of-care inpatient or outpatient investigation	Quickly performed as a point-of-care inpatient or outpatient investigation	More time-consuming, delays with arranging theatre time and a multi-disciplinary team
Very low risk of complications: haemorrhage, nerve injury, and pneumothorax are reported	Low risk of complications: haemorrhage, nerve injury, and pneumothorax are reported	Low-to-moderate risk of complications than CNB and FNA: haemorrhage, nerve injury, pneumothorax, wound infection, and scarring
Can be performed by lower cadre of staff	Required trained operators and can be performed by a lower cadre of staff	Required trained operators and can be performed by a lower cadre of staff
For evaluation of solid organ cancer and recommended with Xpert TB/RIF Ultra as first-line in TB endemic areas	Alternative investigation for LAP in high-risk patients for theatre and deep-seated LNs	Technique recommended for the investigation of LAP, used for small LAP
Inexpensive technique, few resources required: Utilises readily available needles and syringes, with or without an ultrasound	More resources required, but less than a SEB: Utilises a Magnum BARD® gun, or single-use biopsy gun, with or without an ultrasound	More resources required than FNA and CNB: Utilises theatre space, and surgical equipment, with or without an ultrasound
Local anaesthetic	Local anaesthetic	Local anaesthetic

*FNA *Fine needle aspirate, *CNB/USCNB *Core needle biopsy/Ultra-sound guided CNB, *SEB *Surgical excision biopsy, *LAP *Lymphadenopathy, *TB *Tuberculosis, *LN* Lymph node

This study addresses an important gap in the literature: the lack of cost data comparing different diagnostic pathways for LAP in tuberculosis- and HIV-endemic settings. We evaluate the costs of individual diagnostic procedures and clinical pathways, with a focus on a CNB-centred approach recently proposed for earlier lymphoma diagnosis (Antel 2021). These findings may inform policy and budget planning in similar resource-constrained contexts.

## Methods

### Setting and target population

This provider-perspective comparative cost analysis study was conducted at Groote Schuur Hospital (GSH) in Cape Town, a public sector tertiary level hospital in the Western Cape, which receives referrals from primary and secondary facilities in both the private and public sectors. Cross-sectional data were collected from three clinics within GSH. Two surgical clinics were included - the surgical ‘lumps and bumps’ clinic, the ‘acute care clinic’ – both perform SEB as part of routine diagnostic workup for LAP and operate once a week for four hours. Patients requiring further management are followed up at their referring centre. Those with benign SEB results are informed telephonically by the surgeon, and no further intervention is arranged. The third clinic - the ‘Rapid Access LymphAdenopathy Clinic’ (RADLAC) - is a diagnostic research clinic focused on establishing the “Best Care” pathways for LAP investigation in a tuberculosis- and HIV-endemic setting. The RADLAC clinic operates for two hours a week and performs FNAs and CNBs for patients referred with unexplained LAP. Patients with LAP not amenable to CNB, or those with non-LN masses, are referred to the surgical ‘lumps and bumps’ clinic for SEB. The RADLAC dataset includes a previously published prospective cohort of 99 patients referred for unexplained LAP [16]. For this analysis, 83 outpatients were included. Sixteen inpatients were excluded as the diagnostic pathways and associated costs differ substantially due to broader hospital related expenditures, including ward care, and management of acute illnesses or comorbidities.

### Costing methods

The cost analysis combined a bottom-up and top-down costing approach from a healthcare provider perspective. Costing data were collected retrospectively and included data from 2012 to 2021. Annual and unit costs were estimated in South African Rands (ZAR) using 2025 as the base year. Subsequently, all costs were converted to United States Dollars (USD) (US$ 1 = R 18,26, average estimate between January and February 2025) [37]. The average consumer price index (CPI) for the year was applied to inflate the cost when required [38]. The data were collected from the three clinics performing procedures for LAP investigations through on-site and online unstructured interviews with medical, support, and administrative staff.

### Bottom-up costing

Capital costs (building space, furniture, and equipment) and the recurrent costs (personnel, consumables and medication, overheads and maintenance, and laboratory and radiology tests) were determined utilizing the ingredient-based (bottom-up) costing approach. The bottom-up costing amounts were obtained through unstructured interviews.

The building space utilised was estimated by manually measuring the area used for the procedures and apportioned to the procedures based on the time utilised. The cost of the building replacement value per square meter (R 40 468/US$ 2,211) was evaluated using the Order of Magnitude Estimator for new hospitals, used by the Department of Health Infrastructure Unit tool to estimate budgets and assist with new infrastructure planning. A different amount per square meter (R 48 222/US$ 2 635) was used for theatre space. The value per square meter was estimated and multiplied by the area used per square meter [39]. The estimated building and furniture cost was apportioned to procedures based on the time utilised by the clinics and multiplied by the number of patients attending the clinics for the procedure per month and year.

The prices were estimated by averaging the quotation costs received from pharmaceutical companies, the direct expenditure from GSH, and market value costing (three costing averages where possible) from online sources. The capital costs represent an upfront expenditure that depreciates over time and does not always appear on annual reports [40]. Capital items were annualised to adjust for opportunity and depreciation costs by using a 3% rate to allow for international comparison as recommended by the WHO and Global Health Cost Consortium [41, 42]. The estimated lifetimes were 30 years for the building, ten years for furniture, and five years for smaller equipment [41, 43].

To calculate the total cost of the consumables per procedure, we obtained their utilisation rate per procedure through unstructured interviews and multiplied the average cost per unit price. The annual cost was determined by multiplying the total cost of the consumables utilised per procedure by the number of patients attending the clinic (single visit) per year.

The list of investigations (laboratory and radiology testing) was obtained from the unstructured interviews and the existing RADLAC cohort of 83 patients undergoing outpatient diagnostic evaluation for LAP at RADLAC between November 2017 and October 2018. The price list for all laboratory investigations was obtained from the National Health Laboratory Services (NHLS) and utilised in this work, besides the urine Lipoarabinomannan assay (urine-LAM). The urine-LAM price was obtained from a national private laboratory as the price was not available from NHLS during the study. The chest x-ray price was telephonically obtained from two private sector radiologists, where patients pay a fee-for-service rate or an insured rate. The average cost of a single-view x-ray was used as part of the series of baseline tests to exclude tuberculosis.

### Costing of personnel time

Information was obtained through online unstructured interviews initially (due to COVID-19 restrictions) and on-site interviews once permission to regain GSH access was granted. The information included time spent in consultation, on procedures, and additional personnel information (medical, support, and administrative staff), including staff cadre. The annual salaries and grading were obtained from the online published Department of Public Service and Administration document for medical personnel. Annual salaries and grading for support and administrative staff were obtained and inflated to 2025 [44]. The annual salaries were divided by the number of workdays in 2025 and the working hours. Subsequently, these average personnel costs per hour were multiplied by the hours spent per patient consult, follow-up, and procedure to obtain the total personnel cost per patient per procedure.

Costing data were collected through on-site and online interviews with medical, support, and administrative staff (*n* = 15 interviews). As there are few individuals who work directly in the area of LAP diagnosis in GSH, the sample of 15 individuals who were interviewed is almost exhaustive. In order to reduce the recall bias, we asked the interviewees to respond to their previous month’s experience. Estimates were triangulated with hospital procurement records and expenditure reports where possible, to minimise recall bias.

### Top-down costing

A top-down costing method is a systematic approach to calculate indirect costs (not directly associated with the procedure or services) and apportions the total cost to the department or clinic [40]. The overhead costs for the study included utility costs (electricity, water, sewage, and waste removal), administrative costs (telephone, internet, stationery, and printer consumables), cleaning (cleaning materials, cleaning, and laundry), and security costs (security contracts). Additionally, maintenance was included in these overhead costs. The overhead cost was based on total overhead expenditure, assuming all patients utilised the resources equally. Overhead expenditure was divided by the number of patients attending GSH for the year 2017 and estimated using the patient day equivalent (PDE) method of weighing outpatient visits at a third of the inpatient resource use [41, 42].

$$\:PDE\:outpatient=annual\:inpatient\:days\:\times\:3+annual\:outpatient\:visits$$

The overhead cost obtained was placed into relevant procedure categories and summed. The PDE formula was applied, and the total overhead cost was divided by the PDE to obtain the overhead cost per patient, subsequently inflated using CPI to 2025. The 2025 annual overhead cost was determined by multiplying the overhead cost by the number of patients attending the clinics annually, with a single follow-up visit per patient. Training costs were excluded from the expenditure list and not included in the overhead or bottom-up costing.

### Data analysis

Data were collated and analysed using Microsoft Excel for Microsoft 365. Costing was tabulated for comparisons, and pie charts aided in visually analysing the expenditure proportion per procedure.

The number of diagnostic investigations was estimated per the pathway, advised through interviews, and tabulated with their costings. Based on the clinical input via interviews, the average number of FNAs performed before referral for SEB was two. Thus, a table with the pathways and their costs was estimated to reach a definitive diagnosis. The disease prevalence in the sample population was computed over the period of data collection.

The cost of a CNB may vary based on the use of a reusable or disposable automatic biopsy gun and/or ultrasound. A simple two-way sensitivity analysis was conducted to assess the impact on the CNB cost. The costs associated with using a disposable gun (Achieve automatic biopsy needle) and a CNB with or without an ultrasound were compared to using a reusable Magnum BARD^®^ gun with ultrasound (baseline). The procedure cost was also varied based on the cadre of staff performing the procedure, using a simple one-way sensitivity analysis. The caveat being that the time of the procedure was not altered (it could take a clinician longer or shorter with or without the Magnum BARD ^®^ gun and ultrasound, or depending on their cadre).

## Results

### Baseline characteristics of the study population

Based on unstructured medical staff interviews, approximately 288 outpatients attend the three clinics annually for procedures: 76 FNAs and 90 CNBs were administered at the RADLAC clinic; 144 SEBs were performed at the acute care clinic and surgical ‘lumps and bumps’ clinic.

Table 3 presents the baseline clinical and demographic characteristics of the RADLAC cohort [16, 45]. Of the sample, 54% were female, with a mean age of 45 years. Lymphoma was the commonest diagnosis in males (32%) and the second commonest diagnosis overall (23%) after tuberculosis (27%).

**Table 3:** Demographics and disease prevalence in the RADLAC cohort (*n* = 83)

	Females (*n* = 45)		Males (*n* = 38)		Total (*n* = 83)	
	*N*	%	*N*	%	*N*	%
Diagnosis						
Tuberculosis	12	26.7	10	26.3	22	26.5
Lymphoma	7	15.6	12	31.6	19	22.9
Solid malignancy	10	22.2	6	15.8	16	19.3
Other	16	35.6	10	26.3	26	31.3
Age (years)						
20–29	6	13.3	3	7.9	9	10.8
30–39	13	28.9	15	39.5	28	33.7
40–50	11	24.4	8	21.1	19	22.9
>50	15	33.3	12	31.6	27	32.5

In the RADLAC cohort, 76% (63/83) of patients underwent FNA cytology. Cytology was able to provide a definitive diagnosis in 25% (16/63) of cases overall. Lymphoma diagnosis was suspected from cytology in 13% (2/16) of cases, but was never diagnosed. The first CNB was diagnostic in 88% (57/65) of cases, including two patients with preceding non-diagnostic SEB. The final diagnosis was made with a second CNB for four cases and with an excision biopsy for the remaining four cases. In lymphoma cases, CNB was diagnostic on the first biopsy in 89% (17/19), with repeat CNB and SEB making the diagnosis in the two remaining cases.

### Estimated summary of the procedure cost

The annual cost per outpatient procedure is summarised in Table 4. The FNA was the lowest cost procedure, and SEB the most cost-intensive. For the FNA, CNB, and SEB procedures, recurrent costs accounted for 86, 87, and 89% of the total cost, respectively. Capital costs were higher in SEB as a larger area is utilised for this procedure in our setting. Personnel costs were driven by the medical and surgical registrar salaries for all three procedures and were the most significant contributor to recurrent cost for all three procedures - FNA (72%), CNB (59%), and SEB (48%). The second highest recurrent cost driver was building space for FNA (12%), whereas it was consumables and medication for CNB and SEB (20 and 38% respectively).

**Table 4:** Summary of procedural cost estimate per outpatient FNA, CNB and SEB

	ZAR						US$					
	FNA (Lowest)		CNB (Mid)		SEB (Highest)		FNA (Lowest)		CNB (Mid)		SEB (Highest)	
Capital												
Building	R	169.36	R	184.22	R	329.40	$	9.28	$	10.09	$	18.04
Furniture	R	8.99	R	32.47	R	44.53	$	0.49	$	1.78	$	2.44
Equipment	R	12.01	R	26.16	R	5.11	$	0.66	$	1.43	$	0.28
Total capital	R	**190.36**	**R**	**242.85**	**R**	**379.04**	**$ **	**10.43**	** $ **	**13.30**	** $ **	**20.76**
Recurrent												
Personnel	R	1 005.17	R	1 068.51	R	1 718.44	$	55.06	$	58.53	$	94.13
Consumables and medication	R	62.03	R	365.23	R	1 341.01	$	3.40	$	20.01	$	73.45
Overheads and maintenance	R	130.81	R	130.81	R	130.81	$	7.17	$	7.17	$	7.17
Total recurrent cost	**R**	**1 198.01**	**R**	**1 564.55**	**R**	**3 190.26**	** $ **	**65.62**	** $ **	**85.70**	** $ **	**174.75**
Total cost	**R**	**1 388.37**	**R**	**1 807.39**	**R**	**3 569.31**	** $ **	**76.05**	** $ **	**99.00**	** $ **	**195.51**

*FNA *Fine needle aspirate, *CNB* Core needle biopsy, *SEB *Surgical excision biopsy

Note: Text is marked as bold for total values

^†^All costs reported for the year 2025

Additional baseline investigations, including blood and radiology testing, were evaluated separately (Table 5). For all three procedures, these tests were conducted during the initial consultation. The total cost varied based on the investigations requested to confirm the diagnosis, but could be as high as US$ 96. The initial laboratory testing on the samples submitted from FNA, CNB and SEBs is shown in Table 6. Investigations for tuberculosis were more comprehensive for CNB and SEB compared to FNA and thus costs were marginally higher (US$ 34 and US$ 65, respectively). Tissue testing (histology) costs US$ 14 for the CNB and US$ 16 for SEB. Additional tests, such as immunohistochemistry, immunophenotyping with flow cytometry, and fluorescence in situ hybridisation, are not included in the costing as these tests are only requested if indicated based on the initial histology assessment and vary significantly depending on the final diagnosis and differential diagnoses needing to be excluded. In contrast, FNA cytology has a base cost of $ 77, and additional testing is also required to obtain a definitive diagnosis for tuberculosis, lymphoma and other cancer cases, as with CNB and SEB. The high cost of cytology testing on FNA, negated the savings on procedural cost, and the total cost for investigation of tuberculosis and malignancy was US$ 179, higher than CNB (US$ 145) and lower than SEB (US$ 244). Staining costs depended on the type and number of stains but averaged US$ 330 (ranging from US$ 211 to US$ 422).

**Table 5:** The cost^†^ of baseline laboratory and radiological investigation

Cost of Baseline Investigations				
		(ZAR)		(US$)
Laboratory and radiology				
FBC	R	78.19	$	4.28
DIFF	R	42.87	$	2.35
LDH	R	123.02	$	6.74
U and E	R	114.91	$	6.29
HIV Elisa	R	74.38	$	4.07
CD4 Count	R	244.77	$	13.41
Chest x-ray single view	R	696.05	$	38.13
Total	**R**	**1 374.19**	** $**	**75.27**
Additional test				
Urine LAM	R	370.00	$	20.27

*FBC* Full blood count, *DIFF *Differential white cell count, *LDH* lactate dehydrogenase, *U and E *Urea and electrolytes, *LAM *lipoarabinomannan

Note: Text is marked as bold for total values

^†^ All costs reported for the year 2025

**Table 6:** Combined procedural and laboratory costs^†^

Cost of Baseline Tests				
Tests ordered		(ZAR)		(US$)
CNB procedure	**R**	**1 807.39**	** $ **	**99.00**
Xpert MTB/RIF Ultra	R	465.75	$	25.45
TB ZN	R	23.39	$	1.28
TB culture	R	101.69	$	5.57
Histology-1 block	R	250.07	$	13.70
Total CNB (Lowest)	**R**	**2 648.29**	** $ **	**145.06**
FNA procedure	**R**	**1 388.37**	** $ **	**76.05**
Xpert MTB/RIF Ultra	R	465.75	$	25.51
FNA cytology	R	1 410.46	$	77.26
Total FNA (Mid)	**R**	**3 264.58**	** $**	**178.82**
SEB procedure	**R**	**3 568.31**	** $**	**195.51**
Xpert MTB/RIF Ultra	R	465.75	$	25.51
TB ZN	R	23.39	$	1.28
TB culture	R	101.69	$	5.57
Histology-2 blocks	R	290.01	$	15.89
Total SEB (Highest)	**R**	**4 450.14**	** $**	**243.76**

*CNB* Coren needle biopsy, *FNA *Fine needle aspirate, *SEB *Surgical excision biopsy

The text is underlined to show the three procedures

Note: Text is marked as bold for total values

^†^ All costs reported for the year 2025

**Fig. 1:**
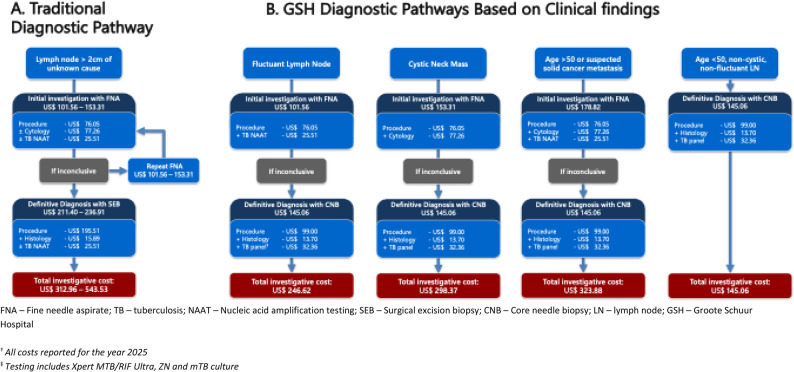
Costed^†^ pathways to diagnoses

In Fig. 1, we present the costing of the observed traditional pathways to diagnosis followed in routine practice as well as the newly implemented pathways at GSH. The traditional pathways reflect a lower initial cost in patients with an initial suspicion of tuberculosis, followed by comprehensive investigation with SEB (pathway 1), in contrast to those with suspected cancer who underwent initial testing with one or the reported average of two FNAs. The GSH pathways document the algorithmic approach followed at the RADLAC clinic.

**Fig. 2:**
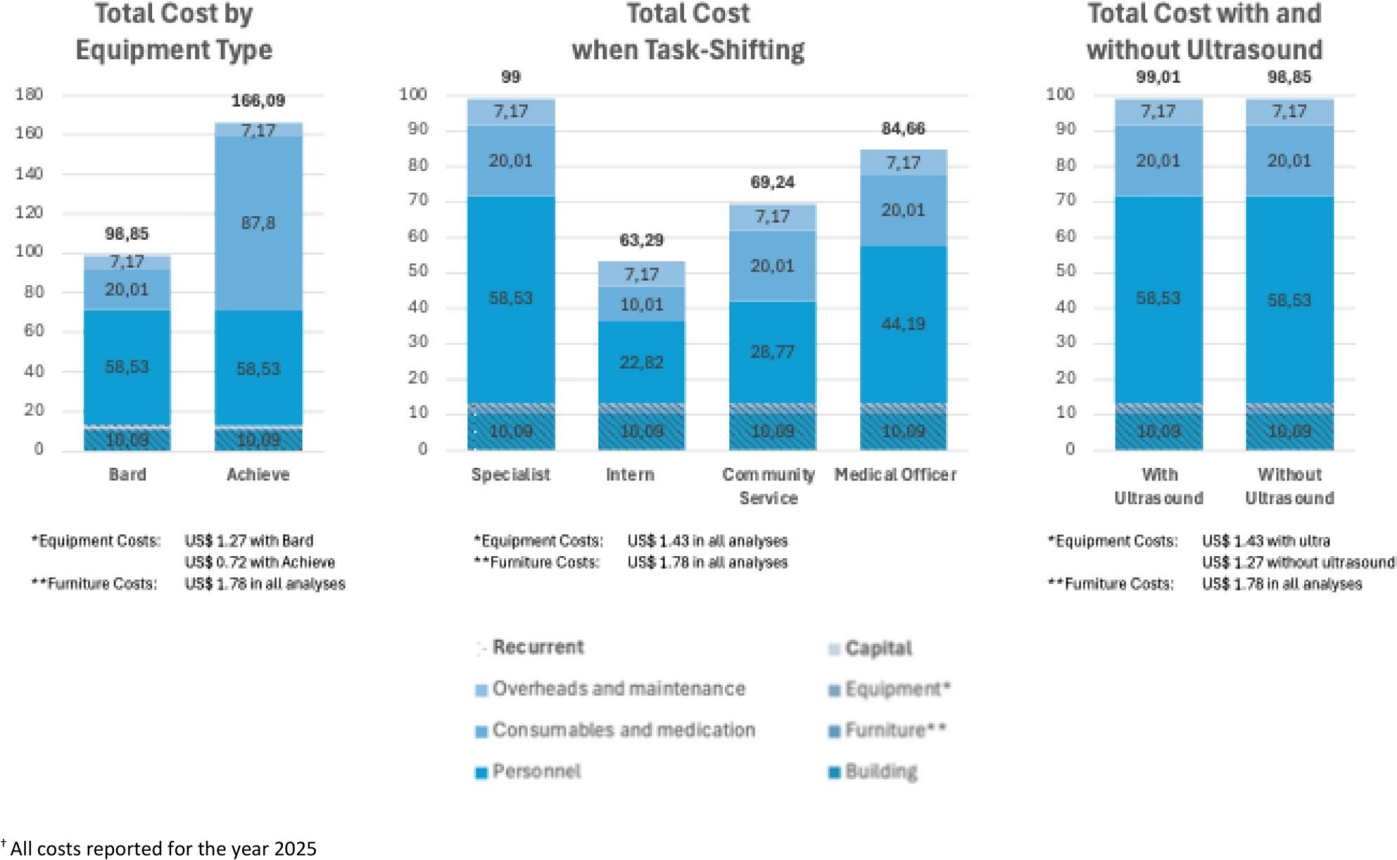
CNB sensitivity analyses

Equipment and consumable costs were varied for the CNB procedure based on ultrasound use and the type of biopsy gun utilised (disposable or reusable), as shown in the sensitivity analysis (Fig. 2). The change in procedural cost when ultrasound was removed was negligible (< US$ 1). The CNB procedure costs are, however, sensitive to the type of biopsy gun utilised, with a single-use biopsy gun (Achieve automatic biopsy needle) estimated to cost 60% more (US$ 67) than reusable or multi-use biopsy guns (Magnum BARD^®^ gun). Utilising a multi-use biopsy gun (Magnum BARD^®^ gun) works out to US$ 13 (R 238) per patient (equipment cost and the needle per patient), compared to US$ 80 (R 1 460) per patient when using the Achieve automatic biopsy needle (i.e., cost of the single-use gun per patient).

Personnel costs were driven by the Clinical Haematology Registrar (FNA and CNB) and Surgical Registrar (SEB) salaries for all three procedures. The estimated time for CNB was 1.03 h, while SEB and FNA were 1.50 and 0.95 h, respectively. When the CNB procedure and consultation were task-shifted to a Medical Officer Intern or a Community Service Medical Officer, or a Community Medical Officer (grade 1–3), the cost per outpatient decreased to between US$ 63 and US$ 85 (see Fig. 2).

## Discussion

The CNB is increasingly acknowledged as a preferred diagnostic modality in numerous clinical settings due to its high diagnostic yield, minimal invasiveness, and practicality in outpatient settings [46, 47]. Offering more robust diagnostic capability compared to FNA cytology, lower cost and improved access compared to SEB, CNB is a potential “Goldilocks” solution. This study presents a detailed cost analysis of the diagnostic investigation of LAP in a tertiary hospital within a tuberculosis-and-HIV-endemic region. By comparing a novel pathway, centred on CNB, to a traditional diagnostic approach in which FNA is followed by SEB, we detail the cost benefit of point-of-care CNB compared both at a procedural level and within patient diagnostic pathways.

The FNA, commonly considered a cost-effective precursor test, can rapidly confirm certain specific diagnoses if appropriate laboratory investigations are selected [16, 48, 49]. For many patients, this time benefit is not realised as limited healthcare services result in long delays between the FNA procedure and appropriate follow-up of results and further investigation [50]. The perceived low-cost is reflected in the cost estimates for each procedure, which confirmed that FNA (US$ 76) was the least costly procedure, followed by CNB (US$ 99) and SEB (US$ 196). However, the up-front cost of cytology meant that initial investigation with FNA was more expensive than a CNB with histology (US$ 179 and US$ 145, respectively), but cheaper than SEB with histology (US$ 244) (see Table 6). Furthermore, the sensitivity of FNA cytology for lymphoma has been shown to be as low as 11%, frequently yielding incomplete, or even inaccurate diagnoses necessitating repeat testing [18, 23, 45]. As the clinical presentations of tuberculosis and malignancies, including lymphoma, frequently overlap, cytology is inadequate, and comprehensive investigation of patients with LAP requires the use of both Ultra and histology.

The CNB, like SEB, provides more tissue than FNA and preserves architecture, allowing comprehensive histological evaluation. This is crucial in lymphoma diagnosis and ensures accurate diagnosis is possible in the majority of cases [23]. A CNB also requires less specialised infrastructure than SEB, contributing to lower capital costs and improved feasibility in decentralised settings. These low barriers to care have been shown to reduce time to diagnosis and potentially will alleviate pressure on theatres and specialised staff performing SEBs [18]. Despite this benefit, the overall costs for the CNB and SEB are comparable, as the time requirements of highly trained medical personnel are equivalent. For this reason, in considering the work-up for patients with LAP or another symptom complex, in whom multiple diagnoses need to be considered, the total cost of the patient’s diagnostic pathway must be considered.

The most significant cost-saving benefit of CNB is seen when it is incorporated in pragmatic diagnostic algorithms, which reduce the number of investigative procedures performed to obtain a definitive diagnosis. As illustrated in Fig. 1, diagnostic pathways that incorporate CNB, rather than SEB, after a single FNA can save up to US$ 66 per patient. However, the limited access to SEB meant that in practice, almost a quarter of patients in the database had more than one FNA before the SEB procedure (traditional pathway 3). This increased the diagnostic pathway cost to US$ 518 and delayed diagnosis. Point-of-care, or rapid access, CNB reduced repeat testing, yielding an additional US$ 102–179 saving per repeat FNA avoided. This benefit is amplified in the investigation of younger patients with non-cystic, non-fluctuant masses, in whom timeous access to CNB allows patients to forego FNA, reducing the total investigative cost to US$ 145. Finally, although not detailed in these pathways, the relatively high cost of additional laboratory and radiological tests that are performed may be reduced if early access to definitive diagnosis with CNB is possible.

Task-shifting could provide significant further cost reductions. The cost of the procedure drove the unit costs of all three investigative procedures, with personnel costs contributing the most (61% on average) for all three procedures. The high personnel cost was associated with the use of medical or surgical registrars to perform the procedures. It has, however, been shown that junior medical personnel can safely obtain high diagnostic accuracy with CNB, and these costs could be reduced through training and task-shifting [51]. The sensitivity analysis confirmed that if medical interns were trained to conduct the procedures, the cost could be reduced by almost 36% (Fig. 2).

Implementing decentralised, non-specialist CNBs could significantly improve the equitable distribution of diagnostic services. Achieving this would require comprehensive training and education to ensure that non-specialist medical staff can accurately identify patients who meet the indications for CNB and perform the procedure safely and competently. Physical opportunity barriers would also need to be addressed, including enabling procurement of the necessary biopsy equipment.

Although task shifting may reduce overall costs, several considerations remain. Non-specialist staff may initially lack the procedural skill and clinical experience required for CNB, necessitating substantial training, ongoing supervision, and quality assurance, all of which require additional resource implications. Furthermore, decentralisation would increase staff responsibilities, and the demand for appropriate equipment and consumables, particularly the correct biopsy needles, requiring reliable procurement systems and sufficient budget allocation.”

This being said, task-shifting and expansion of the RADLAC model to lower levels of facilities would improve equity of access to services. This would be especially beneficial in the rural areas of South Africa.

In addition, a practical recommendation would be to include the diagnosis of haemotological cancers in general, and lymphoma via CNB in particular which could improve the yield and expansion of these services, reduce the time to diagnosis, and result in quicker initiation of treatment.

Our sensitivity analysis also confirmed variation in procedural cost with the selected choice of a biopsy gun, but negligibly for ultrasound, with all other costs held constant. The reusable Magnum BARD^®^ was the preferred biopsy instrument used in practice and was cheaper per outpatient than the Achieve^®^ automated biopsy system (including needles). However, biopsy instrument choice was dependent on consumables and single-use biopsy gun availability.

This study has limitations. Some cost inputs were based on market estimates due to unavailable site-specific prices. Outpatient volumes were used to estimate the procedure costs. Lower patient numbers during COVID-19 possibly affected overhead calculations [52]. Finally, results may not generalise to all settings, as costs depend on local pricing and infrastructure. However, there is transferability of the costs as the groundwork has been laid. The costs are divided into cost components so that new costs can be inserted in place of the costs we have included, such as for new or differing equipment. We found the cost drivers to be the costs of needles and personnel, and so by keeping base costs for all other components, these two aspects (needles and personnel) could be changed or updated. Site-specific factors need to be considered when applying these findings in other settings. For instance, local pricing of biopsy equipment, existing access to ultrasound, and other factors will impact the costing. Nevertheless, we believe our findings can be applied more broadly, especially in LMICs.

### Conclusion and policy recommendations

This study has provided the first cost estimates of FNA, CNB, and SEB procedures at a tertiary facility in South Africa, and the alternative CNB pathway we describe has positive budgetary implications for tertiary hospitals in HIV-and-tuberculosis-endemic areas. The findings demonstrate that CNB offers a higher diagnostic yield than FNA cytology, particularly for lymphoma, which reduces the need for repeat procedures and surgical referral. Improved diagnostic performance, coupled with the lower total investigative cost of CNB, supports the early use of CNB in the diagnostic pathways of patients with unexplained LAP.

The feasibility of point-of-care CNB, its compatibility with task-shifting, and reduced reliance on surgical infrastructure make it especially suitable for implementation in resource-constrained settings. We recommend that health systems integrate CNB into national diagnostic algorithms for LAP, with an emphasis on decentralised access at district and regional levels. Investment in CNB training, procurement of affordable biopsy equipment, and support for clinical task-shifting could enhance diagnostic equity, reduce delays in initiating treatment for tuberculosis and lymphoma, and alleviate pressure on specialist surgical services. These strategies align with broader health system goals of cost containment, timely diagnosis, and improved outcomes in high-burden, low-resource environments.

## Data Availability

The datasets used and/or analysed during the current study are available from the corresponding author on reasonable request.
